# Comparison of Success Rates in Transabdominal Vesicovaginal Fistula Repair With and Without Omental Flap Interposition: A Retrospective Cohort Study

**DOI:** 10.7759/cureus.104802

**Published:** 2026-03-06

**Authors:** Zabish Mehmood, Anam Ghaffar, Saad Ali Khan, Hina Sajjad, Muhammad Muzammil Saqlain, Azka Nayab, Filza Fatima

**Affiliations:** 1 Urology, Sir Ganga Ram Hospital, Lahore, PAK; 2 General Surgery, Jinnah Hospital, Lahore, PAK; 3 Urology, Jinnah Hospital, Lahore, PAK; 4 Obstetrics and Gynaecology, Shalamar Hospital, Lahore, PAK; 5 Endocrinology, Shalamar Hospital, Lahore, PAK; 6 Pathology, M. Islam Medical and Dental College, Lahore, PAK; 7 General Surgery, Pakistan Navy Ship (PNS) Shifa Hospital, Karachi, PAK

**Keywords:** fistula closure, omental flap, transabdominal repair, urinary incontinence, vesicovaginal fistula

## Abstract

Introduction

Vesicovaginal fistula (VVF) is an abnormal communication between the urinary bladder and vagina, resulting in continuous urinary incontinence. Common causes include iatrogenic injuries during obstetric and gynecological surgeries, pelvic malignancies, and radiotherapy.

Objective

To compare the success rate of transabdominal VVF repair performed with and without interposition of a pedicled omental flap.

Methodology

This retrospective study was conducted at Sir Ganga Ram Hospital, Lahore, Pakistan, by reviewing medical records from August 2023 to August 2025. A total of 60 patients with VVF were included. Patients were divided into two groups based on the surgical technique performed: those who underwent repair with an interposition omental flap (Group A, n=30) and those who underwent repair without an interposition omental flap (Group B, n=30). Treatment success was defined as complete fistula closure without urinary leakage, assessed at three months postoperatively.

Results

Successful closure was achieved in 29/30 (96.7%) patients in the Group A compared to 22/30 (73.3%) in the Group B (χ²=6.38, p=0.011). Age did not significantly influence treatment outcomes. However, fistula size and operative duration were significantly associated with surgical success. Large-sized fistulae in the Group B were associated with lower success rates (χ²=16.20, p=0.001), and procedures lasting >120 minutes had reduced success in the Group B (χ²=6.44, p=0.011).

Conclusion

The use of an interposition omental flap significantly improves the success rate of transabdominal VVF repair. Incorporating a vascularized omental graft enhances surgical outcomes and reduces failure rates.

## Introduction

Vesicovaginal fistula (VVF) is an abnormal connection between the urinary bladder and the vagina that results in continuous urinary incontinence. It is the most common acquired fistula of the urinary tract [1]. Recognized causes most commonly include iatrogenic injury during obstetric, gynecological, or urological surgery [2], as well as local tissue damage or invasion secondary to pelvic malignancy or radiotherapy [3]. VVF has a significantly devastating impact on a patient's quality of life, often leading to social isolation, marital disruption, and increased financial burden due to medical and surgical treatment. According to the World Health Organization (WHO), 50,000-100,000 women worldwide develop obstetric fistula each year, and approximately two million young women live with untreated fistula in Asia and sub-Saharan Africa [4].

Achieving a 100% success rate in VVF repair remains challenging. Early diagnosis and timely surgical repair are essential for successful management [5], and the first attempt at VVF repair is associated with higher chances of success [6]. Several surgical approaches are available for VVF repair; however, there is no consensus regarding the optimal technique. The choice of approach generally depends on the characteristics of the fistula and the surgeon's experience [7]. Interposition flaps are used during repair to obliterate dead space, enhance vascularity, and reinforce the repair site [8].

Several studies have evaluated the outcomes of the transabdominal approach with and without an interposition flap. In a case series study, Tariq et al. compared the success rates of VVF repair with and without interposition of an omental flap [9]. The success rate was 96.0% in the transabdominal approach with the interposition omental flap group and 84.0% in the group without an interposition flap. The study concluded that large-sized VVF located at the trigonal or supratrigonal region should be repaired through the abdominal route with an interposition omental flap to achieve optimal results [9]. In a descriptive case series study, Ch et al. evaluated the role of the transabdominal approach with omental graft in VVF repair and reported a success rate of 97.2%. The authors concluded that successful management of VVF depends upon the judicious use of an omental graft with minimal recurrence rates [10].

In a quasi-experimental study, Ashraf et al. assessed the outcomes of VVF repair via the transabdominal approach with and without the use of an omental patch and reported that repair with an omental patch had a higher success rate compared to repair without a patch (76% vs. 57%) [11]. In a retrospective study, Ghosh et al. compared laparoscopic and open abdominal approaches for VVF repair. The comparison showed significantly lower mean blood loss (58.69 mL vs. 147.30 mL; p<0.001), shorter hospital stay (4 vs. 13 days; p<0.001), and reduced analgesic requirement (261.53 mg vs. 617.30 mg; p<0.001) in the laparoscopic group. However, VVF repair was successful in all patients in both groups [12]. In another retrospective study, Ojewola et al. reported that transabdominal VVF repair achieved excellent results in women with previously failed repairs and suggested that the transabdominal approach may be considered as the primary option in such cases [13].

Despite high reported success rates for transabdominal VVF repair, the routine use of interposition flaps remains debated. Some surgeons advocate mandatory use of a vascularized omental flap to reinforce the repair, particularly in larger or complex fistulas, while others reserve flap interposition for recurrent or high-risk cases, citing increased operative time and technical complexity. Although several case series have reported favorable outcomes with omental graft placement, comparative data remain limited and inconsistent, particularly in resource-constrained settings.

The underlying hypothesis of this study is that incorporation of a vascularized omental interposition flap during transabdominal VVF repair significantly increases the likelihood of successful fistula closure compared to repair without a flap. The omentum's rich vascularity, lymphatic drainage, and immunologic properties may enhance tissue healing, reduce tension on the suture line, and provide a mechanical barrier between the bladder and vagina, thereby minimizing recurrence. However, whether these theoretical advantages translate into consistently superior clinical outcomes remains an area of ongoing clinical discussion.

Objective

To compare the success rate of transabdominal VVF repair performed with and without interposition of a pedicled omental flap.

## Materials and methods

Study design and setting

This retrospective comparative study was conducted at Sir Ganga Ram Hospital, Lahore, Pakistan, from August 2023 to August 2025. A total of 60 patients who underwent transabdominal VVF repair during the study period were included. Medical records were reviewed, and patients were divided into two groups based on the surgical technique performed: those who underwent repair with an interposition omental flap (Group A, n=30) and those who underwent repair without an interposition omental flap (Group B, n=30). Non-probability consecutive sampling was used.

Inclusion and exclusion criteria

The study included female patients diagnosed with VVF according to the operational definition, defined as an abnormal epithelial-lined communication between the urinary bladder and the vagina, resulting in continuous involuntary leakage of urine through the vagina, confirmed on clinical examination and appropriate investigations (e.g., dye test and/or cystoscopy). Eligible participants had one or two fistulas located supratrigonally and were between 18 and 65 years of age. Patients were excluded if the VVF was secondary to malignancy or radiotherapy, if it was a recurrent fistula, or if there was a concomitant ureterovaginal fistula (UVF). Additionally, patients with infratrigonal VVF were not included in the study.

Data collection

Ethical approval was obtained from the IRB/ERC of Fatima Jinnah Medical University/Sir Ganga Ram Hospital, Lahore. Due to the retrospective nature of the study, data were collected from hospital medical records using a predesigned proforma. Preoperative, intraoperative, and postoperative variables were documented.

Patients were divided into two groups according to the surgical technique performed: (1) Group A: VVF repair via a transabdominal approach with interposition of the omental flap, and (2) Group B: VVF repair via a transabdominal approach without interposition of the omental flap.

Fistulas were classified according to location, size, and number:

Location: Supratrigonal (above the bladder trigone) confirmed via cystoscopy.

Size: Tiny (<0.5 cm), small (0.5-1.5 cm), medium (1.5-3.0 cm), large (>3.0 cm), and extensive (involving >50% of the bladder wall).

Number: Single vs. multiple fistulas

The decision to use an interposition omental flap was primarily based on surgeon preference and anatomical suitability: Flap was generally preferred for fistulas >1.5 cm, supratrigonal location, or complex/recurrent anatomy. Adequate mobilization of a pedicled omental flap required sufficient length to cover the repair site without tension. If the omentum was thin, adherent, or insufficient in length, flap use was deferred.

All surgeries had been performed by the same consultant surgeon to ensure technical consistency. Demographic and clinical variables, such as age, presenting symptoms, and etiology, were retrieved from patient records.

A Pfannenstiel or infraumbilical incision via the transperitoneal route was used to access the bladder. The bladder was bivalved from the anterior wall to the fistulous site. Bilateral ureteric orifices were stented with 5F Silastic tubes. After encircling the fistula, a plane was developed between the bladder and vagina. The vaginal defect was closed with interrupted 3-0 Vicryl sutures, and the bladder was repaired in two continuous layers. An 18F suprapubic catheter and a 16F perurethral catheter were inserted. Bladder distension was performed to confirm watertight closure, and additional sutures were placed if required. In the interposition flap group, a pedicled omental flap was mobilized and fixed over the vaginal suture line.

Postoperatively, patients were discharged within three to seven days. Immediate postoperative care included placement of 18F suprapubic and 16F perurethral catheters for three weeks, administration of oral antibiotics and anticholinergics (tolterodine 2 mg twice daily), and prescription of stool softeners to minimize straining, along with instructions to abstain from sexual activity for three months. During follow-up, catheters were removed after three weeks, and dye tests or bladder distension were performed to confirm watertight closure. Success was defined as complete closure with no urinary leakage at three months, while patients with persistent leakage were considered repair failures and planned for repeat surgery.

Operative documentation included details of fistula size, location, number, and flap utilization, and all postoperative complications, if present, were systematically recorded.

Statistical analysis

Data were analyzed using Statistical Product and Service Solutions (SPSS, version 24; IBM SPSS Statistics for Windows, Armonk, NY). Numerical variables (e.g., age and operative time) were expressed as mean ± standard deviation, while categorical variables (e.g., symptoms, etiology, VVF size, number, and treatment success) were presented as frequency and percentage. Success rates between the two groups were compared using the chi-square test. Stratification by age, VVF size, and operative time was performed, followed by post-stratification chi-square testing. A p-value ≤ 0.05 was considered statistically significant.

## Results

The mean age of the patients in Group A was 46.46 ± 10.58 years, while in Group B, it was 42.93 ± 10.97 years (p=0.209). Most patients were between 41 and 65 years (20 (66.7%) in Group A vs. 16 (53.3%) in Group B), with no statistically significant difference in age distribution between the groups (Table 1).

**Table 1:** Age distribution and presenting symptoms Values are expressed as mean ± SD or n (%). Comparisons between groups were performed using an independent t-test for continuous variables (age) and a chi-square test for categorical variables (symptoms and age categories). *p ≤ 0.05 was considered statistically significant. SD, Standard Deviation; UTI, Urinary Tract Infection

Variable	Category	Group A (n=30)	Group B (n=30)	Test Value	P-value
Age	Mean ± SD	46.46 ± 10.58	42.93 ± 10.97	t = 1.26	0.209
	18-40 years	10 (33.3%)	14 (46.7%)	χ² = 1.12	0.292
	41-65 years	20 (66.7%)	16 (53.3%)	-	-
Symptom	Passage of gas/stool/pus	5 (16.7%)	4 (13.3%)	χ² = 0.13	0.718
	Foul-smelling discharge	11 (36.7%)	9 (30.0%)	χ² = 0.30	0.584
	Recurrent UTI	19 (63.3%)	16 (53.3%)	χ² = 0.62	0.432
	Irritation/pain	15 (50.0%)	11 (36.7%)	χ² = 1.09	0.297
	Dyspareunia	6 (20.0%)	9 (30.0%)	χ² = 0.81	0.371

Common presenting symptoms included recurrent urinary tract infections (UTIs) (19 (63.3%) in Group A vs. 16 (53.3%) in Group B), irritation or pain (15 (50.0%) vs. 11 (36.7%)), foul-smelling discharge (11 (36.7%) vs. 9 (30.0%)), and dyspareunia (6 (20.0%) vs. 9 (30.0%)). None of these symptoms showed a statistically significant difference between the groups (Table 1).

Abdominal hysterectomy was the leading cause of VVF in Group A 14 (46.7%), whereas vaginal hysterectomy was more frequent in Group B 12 (40.0%). A smaller proportion of cases were attributed to obstructed labor (6 (20.0%) in Group A vs. 4 (13.3%) in Group B) and laparoscopic hysterectomy (3 (10.0%) vs. 4 (13.3%)) (Table 2).

**Table 2:** Etiology, size, number of VVF, and operative time Values are expressed as mean ± SD or n (%). An independent t-test was used for continuous variables (operative time), and a chi-square test for categorical variables (etiology, VVF size, number). *p ≤ 0.05 was considered statistically significant. SD, Standard Deviation; VVF, Vesicovaginal Fistula

Variable	Category	Group A (n=30)	Group B (n=30)	Test Value	P-value
Etiology	Obstructed labor	6 (20.0%)	4 (13.3%)	χ² = 1.52	0.471
	Vaginal hysterectomy	7 (23.3%)	12 (40.0%)	-	-
	Abdominal hysterectomy	14 (46.7%)	10 (33.3%)	-	-
	Laparoscopic hysterectomy	3 (10.0%)	4 (13.3%)	-	-
VVF Size	Tiny	2 (6.7%)	1 (3.3%)	χ² = 1.15	0.76
	Small (0.5-1.5 cm)	10 (33.3%)	9 (30.0%)	-	-
	Medium (1.5-3.0 cm)	10 (33.3%)	13 (43.3%)	-	-
	Large (>3.0 cm)	5 (16.7%)	6 (20.0%)	-	-
	Extensive	3 (10.0%)	1 (3.3%)	-	-
Number of VVF	Single	24 (80.0%)	26 (86.7%)	χ² = 0.48	0.488
	Multiple	6 (20.0%)	4 (13.3%)	-	-
Operative Time	Mean ± SD	123.03 ± 25.70	127.17 ± 17.79	t = 0.72	0.472
	≤120 min	15 (50.0%)	13 (43.3%)	χ² = 0.27	0.605
	>120 min	15 (50.0%)	17 (56.7%)	-	-

Regarding fistula size, medium-sized fistulae (1.5-3.0 cm) were the most common in both groups (10 (33.3%) in Group A and 13 (43.3%) in Group B), followed by small fistulae (0.5-1.5 cm). Multiple fistulae were slightly more frequent in Group A 6 (20.0%) compared to Group B 4 (13.3%), although this difference was not statistically significant (Table 2).

The mean operative time was 123.03 ± 25.70 minutes in Group A and 127.17 ± 17.79 minutes in Group B (p=0.472). Half of the patients (15, 50.0%) in Group A and 13 (43.3%) in Group B had operative times ≤120 minutes, while the remaining patients required procedures lasting more than 120 minutes (Table 2).

Treatment success was significantly higher in Group A (29, 96.7%) compared to Group B (22, 73.3%) (p=0.011). Age did not significantly influence closure rates. However, younger patients (18-40 years) in Group B demonstrated comparatively lower success 10 (71.4%) than those in Group A 10 (100%) (Table 3).

**Table 3:** Treatment success and subgroup analysis Values are expressed as n (%). Comparisons were performed using a chi-square test. *p ≤ 0.05 was considered statistically significant. VVF, Vesicovaginal Fistula

Variable	Category	Group A (n=30)	Group B (n=30)	Test Value	P-value
Overall Success	-	29 (96.7%)	22 (73.3%)	χ² = 6.38	0.011*
Age	18-40 yrs	10/10 (100%)	10/14 (71.4%)	χ² = 3.44	0.064
	41-65 yrs	19/20 (95.0%)	12/16 (75.0%)	χ² = 2.91	0.085
VVF Size	Tiny/Small	12/12 (100%)	10/10 (100%)	-	-
	Medium	10/10 (100%)	12/13 (92.3%)	χ² = 0.80	0.37
	Large	5/5 (100%)	0/6 (0%)	χ² = 21.6	0.001*
	Extensive	2/3 (66.7%)	0/1 (0%)	χ² = 1.78	0.248
Operative Time	≤120 min	15/15 (100%)	13/13 (100%)	-	-
	>120 min	14/15 (93.3%)	9/17 (52.9%)	χ² = 6.53	0.011*

Fistula size showed a significant association with treatment outcomes. All large fistulae in Group A healed successfully, whereas none in Group B achieved closure (p=0.001). Medium-sized fistulae demonstrated high closure rates in both groups (Table 3).

Operative time also influenced outcomes. Both groups achieved 100% closure when the operative time was ≤120 minutes. However, when operative time exceeded 120 minutes, the success rate declined to 9 (52.9%) in Group B compared to 14 (93.3%) in Group A (p=0.011) (Table 3).

## Discussion

In this study, the majority of patients had small- to medium-sized VVF. The success rate of repair was not significantly affected by the age of patients; however, it was significantly lower in cases with large-sized fistulas (p=0.001). Similarly, prolonged operative time was associated with reduced success (p=0.011). Our study identified abdominal hysterectomy and obstructed labor as the leading causes of VVF. Overall, repair with omental flap interposition resulted in higher success rates compared to repair without a flap.

A local study involving a cohort of 17 patients reported that hysterectomy was the most common cause of VVF, followed by obstructed labor, consistent with the findings of the current study. Among their cohort, five patients presented with recurrent fistulas, while the remaining cases were primary. All patients achieved successful repair with no postoperative urinary leakage [14]. These results support the beneficial role of interposition grafts. In our study, the success rate was 96.7% with omental graft interposition, compared to 73.3% without graft [15].

Another study conducted in Lahore compared omental interposition to the placement of peri-vesical fat between the repaired bladder and vagina. Hysterectomy was the most frequent cause of fistula formation in over half of the patients, which aligns with our patient cohort. In the pedicled omental graft group, recurrence occurred in only 2% of patients, whereas no recurrence was noted in the peri-vesical fat reinforcement group during 12 months of follow-up. No perioperative complications, such as wound infection, were reported [16]. Both this study and ours had similar patient characteristics, etiologies, and outcomes.

A separate study conducted in Punjab, Pakistan, evaluated the repair of high-lying complicated fistulas located at or above the bladder trigone. Most of these fistulas were greater than 3 cm in diameter, with only 16% caused by obstetric complications and the remainder resulting from surgical interventions such as hysterectomy. In patients who underwent repair with omental interposition, the failure rate was 4%, corresponding to a 96% success rate, comparable to the 96.7% success rate in our study. In contrast, failure occurred in 16% of patients without graft placement, similar to the 26.7% failure observed in our no-flap group [17-19].

These findings are consistent with previous studies supporting the use of vascularized interposition flaps, such as omental or Martius flaps, in VVF repair. The improved outcomes observed with omental interposition may be attributed to its rich vascular supply, effective lymphatic drainage, and immunologic properties. The omentum promotes neovascularization, provides a mechanical barrier between the bladder and vagina, reduces tension at the suture line, and minimizes the risk of recurrent fistula formation. These biological advantages likely explain the markedly higher success rate observed in the flap group, particularly in larger and more complex fistulas. Acting as a biological barrier, the omentum reduces recurrence risk through its abundant macrophage content and enhanced local vascularity, thereby facilitating optimal tissue healing. The near-universal success observed in our flap group further reinforces this well-established surgical principle [20,21].

From a clinical perspective, these findings suggest that omental interposition should be strongly considered, particularly in large or complex supratrigonal fistulas. Given the significantly lower failure rates observed, routine use of a vascularized interposition flap may optimize surgical success, especially in resource-limited settings where repeat surgeries carry a substantial socioeconomic burden. Despite these encouraging findings, several limitations should be acknowledged. The sample size was relatively small, and the follow-up period was limited to three months, precluding assessment of long-term outcomes, including late recurrence. This was a single-center study performed by a single surgeon; although this ensured technical consistency, it may limit generalizability. The retrospective design introduces potential selection bias, and certain confounding factors - such as fistula size, complexity, intraoperative decision-making, and tissue quality - could not be fully controlled. Additionally, multivariable adjustment was not performed, which may affect the interpretation of observed associations. Future multicenter studies with larger cohorts and extended follow-up are warranted to validate these findings and to more comprehensively evaluate the long-term benefits of omental flap interposition in VVF repair.

## Conclusions

In this retrospective study, repair of VVF with an omental interposition flap was associated with a higher success rate compared to repair without a graft. While patient age did not appear to significantly affect the outcomes, fistula size and prolonged operative time were associated with lower success rates. These findings suggest that the use of an interposition graft may improve surgical outcomes, although prospective studies are needed to confirm these associations.
